# Global, regional and national levels and trends of preterm birth rates for 1990 to 2014: protocol for development of World Health Organization estimates

**DOI:** 10.1186/s12978-016-0193-1

**Published:** 2016-06-17

**Authors:** Joshua P. Vogel, Saifon Chawanpaiboon, Kanokwaroon Watananirun, Pisake Lumbiganon, Max Petzold, Ann-Beth Moller, Jadsada Thinkhamrop, Malinee Laopaiboon, Armando H. Seuc, Daniel Hogan, Ozge Tunçalp, Emma Allanson, Ana Pilar Betrán, Mercedes Bonet, Olufemi T. Oladapo, A. Metin Gülmezoglu

**Affiliations:** UNDP/UNFPA/UNICEF/WHO/World Bank Special Programme of Research, Development and Research Training in Human Reproduction (HRP), Department of Reproductive Health and Research, World Health Organization, Avenue Appia 20, Geneva, Switzerland; Maternal Fetal Medicine Unit, Department of Obstetrics and Gynaecology, Siriraj Hospital, Mahidol University, Bangkok, Thailand; Department of Obstetrics and Gynaecology, Faculty of Medicine, Khon Kaen University, 123 Mitraparb Road, Amphur Muang, Khon Kaen, 40002 Thailand; Center for Applied Biostatistics, Sahlgrenska Academy, University of Gothenburg, Gothenburg, Sweden; Department of Biostatistics and Demography, Faculty of Public Health, Khon Kaen University, Khon Kaen, Thailand; Information, Evidence and Research (IER), World Health Organization, Avenue Appia 20, Geneva, Switzerland

**Keywords:** National, Regional, Global, Preterm birth, Estimates, Trends

## Abstract

**Background:**

The official WHO estimates of preterm birth are an essential global resource for assessing the burden of preterm birth and developing public health programmes and policies. This protocol describes the methods that will be used to identify, critically appraise and analyse all eligible preterm birth data, in order to develop global, regional and national level estimates of levels and trends in preterm birth rates for the period 1990 – 2014.

**Methods:**

We will conduct a systematic review of civil registration and vital statistics (CRVS) data on preterm birth for all WHO Member States, via national Ministries of Health and Statistics Offices. For Member States with absent, limited or lower-quality CRVS data, a systematic review of surveys and/or research studies will be conducted. Modelling will be used to develop country, regional and global rates for 2014, with time trends for Member States where sufficient data are available. Member States will be invited to review the methodology and provide additional eligible data via a country consultation before final estimates are developed and disseminated.

**Discussion:**

This research will be used to generate estimates on the burden of preterm birth globally for 1990 to 2014. We invite feedback on the methodology described, and call on the public health community to submit pertinent data for consideration.

**Trial registration:**

Registered at PROSPERO CRD42015027439

Contact: pretermbirth@who.int

## Background

### Background, rationale, aims and objectives

Preterm birth (PTB) is defined by WHO as all births before 37 completed weeks of gestation [1]. Preterm neonates are at an increased risk for a wide range of short- and long-term respiratory, infectious, metabolic and neurological morbidities, with higher risks of adverse outcomes seen at lower gestational ages [2, 3]. Of the estimated 6.3 million children under 5 who died in 2013, 15.4 % (0 · 965 million, uncertainty range 0 · 615–1 · 537 million) were due to complications of preterm birth; it is the leading cause of death amongst neonates (death in the first 28 days of life) [4].

The WHO estimates are an important resource for assessing the burden of preterm birth at global, regional and national levels, and how that burden is changing over time. Also, they aid development and implementation of health policies, inform resource allocation in health systems, and can be used to assess the impact of interventions. The estimates are also an important tool in raising awareness of preterm birth as an important global public health issue. Two systematic analyses of preterm birth estimates have been published previously [5, 6]. The most recent estimates were published in 2012 (covering data for the period 1990–2010) by Blencowe and colleagues [6]. They estimated that 14 · 9 million babies (uncertainty range 12 · 3–18 · 1 million) were born preterm in 2010, accounting for 11 · 1 % of all live births worldwide. In the 65 countries with reliable time trend data for preterm birth, 62 countries had increasing rates from 1990 to 2010. However, these preterm birth estimates now require updating, in light of new data and continued refinements in statistical modelling methods.

The aim of this study is to develop national, regional and global estimates of preterm birth for all WHO Member States, for the period 1990–2014. This protocol describes the methods that will be used to identify and analyse all eligible data on preterm birth.

The objectives are:

1. To conduct a systematic review of all available data on preterm birth rates;
2. To critically appraise and synthesize eligible data, and conduct modelling to develop estimates of levels and trends of preterm birth rates at national, regional and global levels; and
3. To disseminate preterm birth estimates, in order to inform WHO Member States and other entities in the development of public health programmes and policies.

### Concepts and definitions

The International Statistical Classification of Diseases and Health Problems, 10^th^ revision (ICD-10) [7] uses the WHO definition of preterm birth, namely: “All births before 37 completed weeks of gestation or fewer than 259 days since the first day of a woman’s last menstrual period” [1]. The WHO definition does not define a lower gestational age limit for reporting; ICD-10 advises inclusion of all live births (regardless of gestational age).

Preterm birth can be further sub-divided based on gestational age:

- Extremely preterm (<28 completed weeks of gestation)
- Very preterm (28 - <32 weeks completed weeks of gestation)
- Moderate preterm (32 - <34 completed weeks of gestation)
- Late preterm birth (34 - <37 completed weeks of gestation)

WHO recommends reporting the preterm birth rate using the following indicator (Table 1), which will be the primary outcome for these estimates.

**Table 1:** WHO preterm birth rate indicator

number of liveborn preterm births (singleton or multiple)	
number of live births (singleton or multiple)	

### Factors affecting standardization, measurement and comparison of the live preterm birth rate

Preterm birth includes both spontaneous preterm birth and provider-initiated preterm birth [8]. Spontaneous preterm birth includes both women in spontaneous preterm labour with intact membranes, and women with preterm prelabour rupture of membranes. Provider-initiated preterm birth includes women in whom the preterm delivery is initiated (either by induction of labour or caesarean section) for maternal or fetal indications. While it is often stated that approximately 20–30 % of preterm births are provider-initiated [8, 9], this can vary greatly between countries, and is significantly lower in many lower-income countries [10].

The pathophysiology of spontaneous preterm birth is not fully understood and several pathways have been identified [8]. While many socio-demographic, nutritional, medical, obstetric, biological and environmental factors increase the risk of preterm birth, many women who deliver preterm who do not have a clear risk factor.

Developing estimates is further complicated by several factors that can impede accurate preterm birth data measurement, estimation and comparison:

- The risk of preterm birth can be higher in some disadvantaged sub-populations (including poor, uneducated, rural-dwelling women or other minorities) where data collection may be more limited and/or facility-based births are less common;
- Misclassification of live births, stillbirths and neonatal deaths can also impact on accurately recording the liveborn preterm birth rate. As the risk of stillbirth is higher in earlier gestation, measuring the liveborn preterm birth rate can underestimate the total preterm birth burden; [11]
- National differences in the definition of preterm birth (for example, using live births or total births as the denominator, and different gestational age thresholds for defining preterm birth cases), and the relevant lower gestational age boundary for registration can complicate comparisons;
- Similarly, countries with a lower gestational age boundary for birth registration (i.e. viability) will capture a larger absolute number of births and preterm births. However, this can also potentially improve preterm birth registration rates at higher gestational ages [6].
- Gestational age (GA) estimation error is also an important factor. Generally, the later in pregnancy a GA estimate is made, the wider the uncertainty of that estimate. Routine early pregnancy ultrasound with fetal biometric measurements is considered the “gold standard” for gestational age assessment [12]. However, other methods such as calculation from date of last menstrual period (LMP), symphysis-fundal height measurement, postnatal examination of the newborn, or use of birthweight as a gestational age surrogate are often used in resource-limited settings. Many countries report the use of “best obstetric estimate” of gestational age, using a combination algorithm of ultrasound and LMP [13].

## Methods

### Overview

There are two broad categories of preterm birth data available:

1. Routinely collected birth data, available from national Ministries of Health or statistical offices (civil registration vital statistics); or
2. Data from published research studies

For this study, high-quality, civil registration vital statistics (CRVS) data is the preferred data source. Civil registration is defined by the United Nations as the “continuous, permanent, compulsory and universal recording of the occurrence and characteristics of vital events […] pertaining to a population” [14]. However, for many countries, CRVS data on preterm birth will be incomplete, of poor quality or not available [15]. In countries with absent or limited CRVS data, data from research studies will be required.

While reproductive health surveys may use population-based random sampling and have large sample sizes, they generally rely on maternal recall and knowledge of preterm birth, and hence may have poor accuracy and limited utility. Conversely, facility-based studies, particularly in settings where early antenatal care participation is high and obstetric ultrasound is available, may have greater accuracy in estimating GA and diagnosing preterm birth, however they often have smaller sample sizes and may not be representative of the general population. Consequently, it is difficult to ascertain whether certain data sources or study designs are more useful, or should supersede, other sources. Initially, we intend to be over-inclusive (in terms of study designs) and conduct necessary exploratory analyses of all included data, to explore the quality, representativeness and utility of identified data. This may result in later exclusion of certain data sources.

Following the identification and extraction of relevant data, statistical analysis and modelling will be completed to develop estimates of levels of preterm birth at global, regional and national levels, with trends at national level where sufficient data is available. The methodology and preliminary estimates will then be reviewed by a technical advisory group (TAG) (an independent group of experts in obstetrics, neonatology, statistics and preterm birth research). The TAG will advise if any modifications are required. As per WHO standards in developing official estimates, we will also conduct a country consultation process, whereby WHO Member States are invited to review their preliminary estimates and submit any additional data not identified in the search. From this, final estimates will be developed and disseminated. This protocol has been prepared according to the Preferred Reporting Items for Systematic reviews and Meta-Analyses for Protocols (PRISMA-P) 2015 statement [6].

### Eligibility criteria

For searches of CRVS and study data, the following eligibility criteria will be used:

#### Population

Liveborn neonates (singleton and/or multiple). Data reported using a related or similar definition of preterm will also be included. This includes preterm birth for all births (rather than live births only), singletons only, non-malformed fetuses only, or <36 weeks gestation. Women, pregnancies or newborns of non-generalizable sub-populations (such as those with specific medical or obstetric complications only, women or newborns using speciality services, selected sociodemographic groups, sub-groups based on maternal age or otherwise high-risk or selected populations) will not be included. Studies of low-risk populations only will be included.

#### Setting

Data from national or subnational level (including population-based, community-based and facility-based data) for the 194 Member States of WHO [16] will be considered for inclusion.

#### Study design

Any study design capable of producing usable data on preterm birth will be eligible for inclusion (e.g. surveys, cross-sectional studies, interventional studies) regardless of the context or the setting (e.g. nationwide, facility-based). Conference abstracts will not be included, due to insufficient data.

#### Outcomes

- Primary outcome:
  - ○ Preterm birth rate (in all live births, according to the WHO definition)
- Secondary outcomes:
  - ○ Preterm birth in all births (liveborns and stillborns)
  - ○ Extremely preterm (<28 completed weeks)
  - ○ Very preterm (28 - <32 completed weeks)
  - ○ Moderate preterm (32 - <34 completed weeks)
  - ○ Late preterm birth (34 - <37 completed weeks)
  - ○ Spontaneous and provider-initiated preterm birth (<37 completed weeks)

#### Timeframe 

All available data on preterm birth from 1990 to 2014.

#### Languages

No language restriction will be applied.

#### Sample size

There is no established method for defining a minimum sample size per data source for the development of global estimates. Previous preterm birth estimates have used cut-offs of 50 births [6] or 200 births [5] for inclusion; the 2012 estimates also used a cut-off of data obtained over ≥12 months [6]. For these estimates, a lower limit of 500 births (without time restriction) was selected as a cut-off by consensus of the working group, based on the following rationale:

- Smaller studies may be more susceptible to bias (eg: selection bias);
- Based on the 2012 estimates [6], it is likely that or more covariates will be required to estimate the preterm birth rate. Assuming a minimum of ten preterm birth cases per covariate, 50 preterm birth cases would be needed. Assuming a preterm birth rate of approximately 10 %, this equates to 500 births in total per dataset.

### Classification of countries for assessing reliability and quality of available data

Different global estimates have used different methods of categorizing or stratifying countries on the basis of available data [6, 17, 18], depending on the outcome of interest. For example, previous maternal mortality estimates have used a three-level system for categorizing countries on the basis of the quality of mortality data reporting [18]. However, birth registration levels are generally higher than those of death and cause-of-death registration [19]. Classifying countries on the basis of cause-of-death completeness is probably not an appropriate proxy for the capture of data related to preterm birth.

For the purposes of developing preterm birth estimates, high-quality CRVS data on births should have both reasonably high coverage and completeness, as well as reporting the necessary data to determine the preterm birth rate and the method/s of gestational age assessment. However, lower-quality CRVS data on preterm births may still be useful as an input for statistical models.

The WHO Global Health Observatory reports national estimates of civil registration coverage of births [20]. This country level indicator is derived from assessments of civil registration systems, and/or from population-based household surveys that report on proportion of births registered. The latest World Health Statistics Report (2015) [21] also reports on this indicator (for countries where data is available) based on data from the period 2007 to 2013. This indicator will be used as a proxy for identifying countries likely to have higher-quality CRVS data for preterm births for the period of interest (see Table 2 below). For countries where data is not available, we will consult with relevant WHO technical staff to inform correct categorization.

**Table 2:** Civil registration coverage of births for 194 WHO Member States [21]

Civil registration coverage of births (%)	Number of countries (%)
>80 % coverage	126 (64.9 %)
60 % - ≤80 % coverage	23 (11.9 %)
<60 % coverage	29 (15.0 %)
No data	16 (8.2 %)
Total	194 WHO Member States

We plan to classify all WHO Member States into Groups A, B and C, on the basis of: whether CRVS data is available on preterm birth, the coverage of the civil registration system for births, and the volume of the CRVS preterm birth data available. Hence:

- Group A: Countries with CRVS birth registration coverage is over 80 %, and CRVS preterm birth data is available for > =50 % of years from 1990 to 2014 inclusive;
- Group B: Countries with CRVS birth registration coverage from 60 to 80 %, and/or CRVS preterm birth data is available for <50 % of years from 1990 to 2014 inclusive (or otherwise ineligible for Group A);
- Group C: Countries with CRVS birth registration coverage below 60 % or unknown, and/or no CRVS data for preterm birth is available (or otherwise ineligible for Group A and B).

The overview of this categorization is provided in Table 3. In some instances, expert judgment from WHO technical staff may be used to further inform correct categorization. Countries in Group A will be considered as having high-quality data for preterm birth, and no further searching will be conducted (i.e. CRVS data only will be used for these countries). For countries in Groups B and C, available CRVS data will be used as inputs for statistical models, however further searching (systematic review of available literature) will be conducted for these countries. It is envisaged that some CRVS data may be of such poor quality that it cannot be used, and will be excluded.

**Table 3:** Categories and criteria for Groups A, B and C in WHO preterm birth estimates

		WHO Member States		
		Group A country	Group B country	Group C country
Quality Criteria	Source	Specific criteria		
1. Preterm birth data available	Assess available CRVS data	CRVS data for preterm birth are available, ie: Preterm birth rate is given or can be calculated	CRVS data for preterm birth are available, ie: Preterm birth rate is given or can be calculated	No CRVS for preterm birth available, or CRVS data available but country does not otherwise meet Group A or B criteria
2. Quality of civil registration system for births	Civil registration coverage of births (%)	CRVS coverage for births >80 % [20] (or country can be otherwise classified as having high coverage for birth registration)	CRVS coverage for births is 60 % - <80 % [20]	CRVS coverage for birth data is <60 % or unknown [20]
3. Volume of CRVS data	Assess available CRVS data	• ≥500 births • Commencement date of data collection after 1/1/1990 • End date of data collection is before 31/12/2014 • Number of years where preterm data available is >50 % for the period 1990 to 2014 inclusive (i.e. CRVS data available for at least 13 years)	• ≥500 births • Commencement date of data collection after 1/1/1990 • End date of data collection is before 31/12/2014 • Data for any year/s between 1990 to 2014 inclusive (but does not meet Group A criteria)	• No CRVS data on preterm birth available, or • CRVS data on preterm birth available, but country does not otherwise meet Group A or Group B criteria^*a*^
Data sources for development of estimates				
Data sources:		CRVS data only to be used	Conduct systematic review of published studies to identify additional data sources	Conduct systematic review of published studies to identify additional data sources

^a^Countries that have CRVS data for preterm birth, but CRVS coverage for birth data is <60 % or unknown, will be considered Group C countries

The systematic review of data from Group B and C countries will consider both interventional and observational designs (case-control studies will be specifically excluded) presenting original quantitative data on preterm birth. If intervention studies are included, all arms will be considered for inclusion. However, if the preterm birth rate is significantly different between arms (or the significance is unknown), only the control arm/s will be used.

### Search strategy and classification of countries

CRVS data will be obtained through online searching of national Ministry of Health and national statistical office publications and datasets for every WHO Member State. A review of references from the 2012 WHO preterm birth estimates activity [6] will be used to supplement this search, as will eligible data from the country consultation (see below).

For the systematic review of published studies, the following databases will be searched: Medline, EMBASE, Popline, WHO Global Health Library (including regional and global indexes), CINAHL, PsychInfo, and the Cochrane Central Register of Controlled Trials (CENTRAL). Citations from previous systematic reviews of levels and trends of preterm birth will also be reviewed [5, 6]. A *pro forma* email specifying our analysis objectives and inclusion criteria will be developed and circulated to further identify data. We will contact key stakeholders working in each country and in maternal and neonatal health research networks in countries where little or no data is available, in order to further identify additional unpublished datasets (if available). Given the population size of China, and the lack of a national CRVS data on preterm birth, we will conduct a separate search of Chinese language health and medical databases, using the same protocol.

### Screening, data selection and collection process

Two reviewers will independently screen all citations (title and abstract) identified through the searches, to assess for potential eligibility. In the case of disagreement or where the information is not sufficient for decision on inclusion/exclusion, the article will be included for full text review. Full texts of potentially eligible studies or sources will be retrieved and independently assessed for inclusion by two reviewers. Any discrepancies will be resolved by discussion and consensus by the two reviewers or through consultation with a third reviewer. Where citations are excluded at this step, the reason for exclusion will be documented.

We will develop a standard data extraction form that will be pilot tested against both CRVS and study data on preterm birth. Data extraction from the full text articles will be done using this standard form via an online data management database, completed independently by two reviewers with the results compared. In the event of disagreement, discrepancies will be resolved by discussion and consensus, or by consultation with a third reviewer. All data inclusion and exclusion will be reported according to the PRIMSA checklist.

Extracted data will include: country, data source, design, time period, definition and methods used, range of gestational age used, method of GA assessment, as well as prevalence and incidence data on preterm birth (according to primary and secondary outcomes) and covariate data (see below).

### Assessing quality of data sources

In this review we will potentially include data from a range of study designs. However there is currently no established tool for standardized assessment and comparison of quality across multiple study designs. To this end, the WHO abortion estimates working group developed a data quality assessment tool, with the aim of “systematic, quantitative, and efficient differentiation of studies into different quality levels” (Tuncalp O, personal communication). The quality of data sources was assessed using a five-point checklist, adapted from items within the Strengthening the Reporting of Observational Studies in Epidemiology (STROBE) statement. Items were selected to assess quality in distinct sections of the data source, to be relevant across a range of study designs, and to be manageable in terms of time required to conduct the quality assessment.

This has been reviewed and adapted for use in these estimates. The checklist includes the following five domains:

1. Eligibility criteria for the participants in the study are provided;
2. Method of gestational age assessment (i.e. measurement of the primary outcome) are provided;
3. Characteristics of study participants (e. g. demographic, clinical, social) are provided;
4. The numbers of all outcome events/summary measures are reported;
5. Authors discuss relevant sources of potential bias and/or imprecision in the limitations/discussion.

Each item will be rated as met (1 point) or unmet/not clear (0 point). Hence, for a given data source a maximum score of 5 is possible. Reviewers that extract data will score the data source based on the above criteria. Scores will be imputed by the reviewer into the data extraction spreadsheet. In the case of disagreement, agreement will be reached through consensus, or through engaging a third reviewer.

Once completed, the score distribution will be assessed and reported descriptively. Data sources with the lowest scores will be re-evaluated; we will consider excluding data sources on the basis of low quality. Sensitivity analyses may also be used. These scores will not be used for weighting in modelling.

### Regions

Regions will be defined and reported according to multiple official groupings, including United Nations Regional Groups, WHO Regions, Sustainable Development Goal Regions, World Bank Regions and Income Groups, and other UN agency regional groupings (UNFPA, UNICEF, UNDP).

### Covariates

In countries with little or no preterm birth data available, modelling the preterm birth rate on the basis of covariates will be required. Candidate predictors have been preliminarily identified based on clinical relevance, known risk factors for preterm birth and covariates identified in previous preterm birth estimates. These are:

- Neonatal mortality rate
- Low birthweight rate
- Caesarean section rate
- Multiple birth rate
- HIV prevalence rate (women 15–49 years old)
- Malaria rate (notified cases of malaria per 100,000 population)
- Proportion of women receiving four or more antenatal care visits
- Mean adult female BMI
- Gross Domestic Product (GDP) per capita
- Total fertility rate
- Female literacy rate
- Human Development Index
- Maternal mortality ratio (MMR)
- Adolescent pregnancy rate
- Skilled birth attendance rate
- Facility delivery rate
- Gestational age range used by countries (for live birth and preterm birth registration)

We aim to identify and extract available data on these covariates from individual studies included in the systematic review. This will allow modelling using study-specific covariate values. Additionally, it will permit a better description of the study database, identify potential selection biases (i.e. selected populations) in identified studies, and also facilitate possible sensitivity or secondary analyses (if required). However, it is unlikely that individual studies will contain data on all the covariates identified above. We identified a shortlist of covariates where data is likely to be available, which will be extracted from individual studies:

- Neonatal mortality rate
- Low birthweight rate
- Caesarean section rate
- Adolescent pregnancy
- HIV rate (for the study population)
- Malaria rate (for the study population)
- Proportion of women receiving four or more antenatal care visits

Where study-specific covariate data are not available, national estimates of covariates from the most comprehensive United Nations sources will be used.

## Result

### Statistical analysis and modelling

In the primary analysis all data (regardless of definition of preterm birth) will be used. The WHO definition for preterm birth rate will be the reference definition in the analysis and for the presented results. Some data sources may use alternative definitions of preterm birth, in which case proportionate adjustment of the regression models will be explored.

We hypothesize that the magnitude of associations between covariates and preterm birth are likely to vary by region. Hence, we will develop region-specific models (described below). However, once the database is assembled, we will conduct exploratory analyses to assess whether sufficient data exists to permit development of these region-specific models and if not, a global model will be developed.

For Group A countries, only CRVS data will be included. For Group B & Group C countries, both CRVS and study data will be included. All included data will be analysed as described below (regardless of country group). The predicted preterm birth rate for 2014 at national, regional and global levels will be presented. For those countries with sufficient data, trends will also be presented (see below). All analyses will be conducted using Stata 14.

#### Preparation for analysis

To facilitate the development of models, we will obtain estimates of country level covariates from comprehensive, publicly available United Nations sources. If country covariate data is missing for some countries and/or timepoints, these will be imputed based on separate regression models. Where available, data on selected covariates will also be extracted directly from data sources. If a data source-specific covariate value is available, this will be used preferentially in the models over covariates from national estimates.

#### Phase 1: modelling

In the first phase, the preterm birth outcome will be modelled using a two-level (country and data source) linear mixed regression model, including random country-specific intercept and slope. The model will include time, data source characteristics and covariates. Inclusion of covariates will be based on model fit parameters (BIC, AIC) by removing one covariate at a time and refitting the model. If the model is improved by removing the covariate, the covariate will be excluded. Linearity will be assessed graphically per country. For those regions that contain countries where the relationship between covariates and the preterm birth outcome is clearly not linear, a spline function or log transformation will be considered. In this phase, no preterm birth outcome data will be imputed for missing countries and/or timepoints. Hence, the regional models will only be based on available data. Restricted maximum likelihood estimation (REML) will be used to develop the regional models, and country random effects will be calculated by best linear unbiased predictor (BLUP).

#### Phase 2: prediction of preterm birth rate in 2014

The predicted preterm birth rates in 2014 will be calculated based on the regional models, using country-specific random coefficients and covariate data. Regional and global predictions for 2014 will be calculated based on the national estimates, weighted using number of live births. Predictions for countries providing no preterm birth outcome data will be based on the regional average (i.e. random coefficients assumed to be zero) adjusted according to country covariates. Standard errors for regional and global predictions will be calculated from country level standard error. For countries providing no preterm birth outcome data the standard error will be assumed to be of the same level as the country having the largest standard error in the region.

#### Phase 3: presentation of results

For all countries, regions and globally the 2014 predicted preterm birth rate will be presented together with 95 % confidence intervals. For each country, both the reported and the predicted preterm birth outcome data will be graphed from the first year where data was available to 2014. Both continuous predictions and means over 5 year periods will be presented. For countries where no preterm birth outcome data was identified, only the 2014 predicted value will be presented.

### Country consultation

Following the development of the preliminary estimates, WHO will conduct an official country consultation. WHO Member States (via their focal points) will be invited to confidentially review the methodology and preliminary estimates for their country. They will also be invited to submit additional data that may not have been identified through the searches. If additional data is identified, it will be reviewed and included if eligible. Subsequently, the models will be re-fit including these new inputs.

### Project management

HRP/RHR will lead the technical activities related to data search and synthesis, as well as country consultations, in order to produce updated official WHO estimates. A Technical Advisory Group (TAG) will be established to provide oversight and technical input on the development of these estimates. The TAG will comprise a group of international experts on preterm birth estimates and modelling techniques.

## Discussion

This study will be used to generate estimates of rates and trends of preterm birth at national, regional and global levels for 1990 to 2014. These estimates are a useful resource for public health providers, researchers and policymakers, in order to advance understanding the burden of preterm birth, raise awareness and to better target and evaluate public health programmes and track progress.

We invite feedback on the methodology described, and call on the public health community to submit unpublished yet potentially eligible data for consideration, to WHO via pretermbirth@who.int.

## Abbreviations

BLUP, best linear unbiased predictor; BMI, body mass index; CRVS, civil registration and vital statistics; GA, gestational age; GDP, gross domestic product; HIV, human immunodeficiency virus; HRP, UNDP/UNFPA/UNICEF/WHO/World Bank Special Programme of Research, Development and Research Training in Human Reproduction; ICD, International Classification of Diseases; LMP, last menstrual period; MMR, maternal mortality ratio; PTB, preterm birth; RHR, Department of Reproductive Health and Research; TAG, technical advisory group; UNDP, United Nations Development Programme; UNFPA, United Nations Population Fund; UNICEF, United Nations Children’s Fund; WHO, World Health Organization
